# Whole-Section Tumor Micro-Architecture Analysis by a Two-Dimensional Phasor-Based Approach Applied to Polarization-Dependent Second Harmonic Imaging

**DOI:** 10.3389/fonc.2019.00527

**Published:** 2019-06-19

**Authors:** Riccardo Scodellaro, Margaux Bouzin, Francesca Mingozzi, Laura D'Alfonso, Francesca Granucci, Maddalena Collini, Giuseppe Chirico, Laura Sironi

**Affiliations:** ^1^Physics Department, Università degli Studi di Milano-Bicocca, Milan, Italy; ^2^Department of Biotechnology and Biosciences, Università degli Studi di Milano-Bicocca, Milan, Italy

**Keywords:** second harmonic generation, phasor approach, collagen, cancer, two-photon microscopy, label-free imaging

## Abstract

Second Harmonic Generation (SHG) microscopy has gained much interest in the histopathology field since it allows label-free imaging of tissues simultaneously providing information on their morphology and on the collagen microarchitecture, thereby highlighting the onset of pathologies and diseases. A wide request of image analysis tools is growing, with the aim to increase the reliability of the analysis of the huge amount of acquired data and to assist pathologists in a user-independent way during their diagnosis. In this light, we exploit here a set of phasor-parameters that, coupled to a 2-dimensional phasor-based approach (μMAPPS, Microscopic Multiparametric Analysis by Phasor projection of Polarization-dependent SHG signal) and a clustering algorithm, allow to automatically recover different collagen microarchitectures in the tissues extracellular matrix. The collagen fibrils microscopic parameters (orientation and anisotropy) are analyzed at a mesoscopic level by quantifying their local spatial heterogeneity in histopathology sections (few mm in size) from two cancer xenografts in mice, in order to maximally discriminate different collagen organizations, allowing in this case to identify the tumor area with respect to the surrounding skin tissue. We show that the “fibril entropy” parameter, which describes the tissue order on a selected spatial scale, is the most effective in enlightening the tumor edges, opening the possibility of their automatic segmentation. Our method, therefore, combined with tissue morphology information, has the potential to become a support to standard histopathology in diseases diagnosis.

## Introduction

Histopathology based on excisional biopsy and tissue staining by Hematoxylin/Eosin and ImmunoHistoChemistry (HE and IHC) (1, 2) is the golden standard in clinical diagnostics for accurate and timely diagnosis of cancers. Fluorescence-based techniques have also been exploited to characterize the cellular and subcellular structures, both in healthy and pathological tissues. However, these techniques are characterized by some severe drawbacks: extensive tissue manipulation that can lead to artifacts, expensive and time consuming labeling protocols, lack of 3D sectioning, and, most important, delay in obtaining the diagnosis and interpretative variability (3–5).

In this landscape, label-free microscopy has gained increasing success due to its capability to overcome the above-mentioned issues and to aid pathologists in fast and reliable tumor diagnosis, with potentially great impact on the health of the population and the sustainability of the healthcare systems (6–8). Recently, the intrinsic fluorescence signal coming from intracellular molecules (e.g., NADH, FAD, flavoproteins, lipofuscins, or aminoacids) or tissue constituents, has been exploited to provide diagnostic information on different pathologies (9–11). Moreover, hyperspectral imaging which analyzes the (auto)-fluorescence or reflectance spectral profiles peculiar of each tissue, has become a promising method in diseases evaluation (12–15). Also Coherent Anti-Stokes Raman Scattering (CARS) microscopy, that provides information on the molecular composition of the sample through the Raman spectrum, has been used to identify and characterize the properties of tumors (16–18).

Furthermore, apart from the fluorescence intensity and its spectral features, fluorescence excited state lifetime (19–21) and non-linear scattering properties of the tissues, such as Polarization-resolved Second Harmonic Generation, P-SHG (22–31), have been exploited. These features have been also combined with a phasor-based analysis to extract information on healthy and pathological tissue conditions (32–40) and to identify diseases such as tumor types and their development stages.

Despite the high resolution, the molecular contrast achieved and the reduced scattering, non-linear optical based techniques still suffer the disadvantage of limited penetration depth (typically <1 mm) into tissues and reduced field of view (~500 μm^2^). These limitations are at least partially overcome by optical coherence tomography (OCT) (41, 42) and Photo-Acustic (PA) imaging (43). OCT is a minimally invasive technique, that can be performed in real time and *in situ*, and that offers a high structural sensitivity, with a resolution of 1–10 μm and a penetration depth of 2–3 mm, although it provides unspecific contrast. On the other hand, PA imaging offers a higher penetration depth with respect to OCT, while it suffers from low resolution and low structural contrast.

In parallel to the efforts devoted to improve the image acquisition modes, image analysis tools that can assist pathologists in the characterization of the tissue properties are gathering increasing interest, with the aim to reach real-time automated *in-vivo* cancer diagnosis. User independent algorithms for the automatic extraction of disease features are attracting more and more attention due to their deep implications for resolving pathological cues and increasing the reliability of the results at reduced costs (44–49). Moreover, due to the large amount of acquired data, a dimensionality reduction for the extraction of significant and valuable features describing diseased tissues has to be faced.

By working along the above research lines, we recently developed μMAPPS (Microscopic Multiparametric Analysis by Phasor projection of Polarization-dependent SHG signal), a 2D phasor analysis of the polarization dependent SHG signal collected on images (39) to provide micro-structural information (at pixel level) of the collagen architecture in the tissue extra-cellular matrix (ECM). In fact, collagen molecules suffer remodeling during pathological development that could be exploited for diagnostic purposes (23, 50, 51). Our 2D phasor algorithm provides both the mean orientation angle of the fibrils (θ_F_) and the susceptibility anisotropy parameter (γ, the ratio of off-diagonal to diagonal elements of the susceptibility tensor ${\underline{\underline{\chi }}}^{\left(2\right)}$), converting huge optical dataset in dispersion plots in the Fourier space. In such derived space, the application of a clustering algorithm (52) allows to further reduce the dimension of the data and to recognize automatically tissutal regions sharing similar microscopic behavior without the need to resort to direct feature segmentation.

Our main aim here is to show that the μMAPPS phasor approach can be applied to large tissue sections (few mm in size, comparable with histo-pathology sections) by analyzing them as separate tiles sequentially acquired on an optical microscope. In order to test the possibility to automatically discriminate tissue regions with different collagen organization in large histopathology sections, we develop a set of parameters defined in the phasor space (called hereafter p-parameters) that work on the θ and γ distributions at a mesoscopic level (75–150 μm regions of interests instead of single pixels).

We report a proof-of-concept of the application of this method to the automatic segmentation of tumor tissues surrounded by a skin layer in histopathology sections. We performed a two levels analysis: at a first level, the tumor and skin area were manually separated by an expert and analyzed on a global scale, while in the second one our algorithm was recursively applied on sequential regions of interest (ROI) of increasing size encompassing the entire section, in order to automatically quantify the local heterogeneity of the tissues organization. As a proof of principle of the applicability of μMAPPS to histo-pathological analysis, we investigated two cancer models, obtained from colon carcinoma CT26 cells and breast cancer 4T1 cells implanted in mice.

The p-parameters whose efficacy in highlighting the tumor edges was tested here are: the mean Cluster Elements Ratio (ratio between the number of elements in each retrieved cluster and in the most populated cluster, CER), the number of clusters (N_C_), and the “fibril entropy” (S). This latter parameter, which describes the tissue order on a selected spatial scale, shows to be, at least at this proof-of concept stage, the most effective and significant one to automatically discriminate different collagen microarchitectures and enlighten the tumor edges (skin-tumor boundaries).

## Materials and Methods

### Two-Photon Microscopy Set-Up

The optical setup is built around a confocal scanning head (FV-300, Olympus, Japan) mounted on an upright optical microscope (BX51, Olympus, Japan) and coupled to a fs-pulsed Ti:Sa laser (690–1,040 nm, 80 MHz repetition rate, Mai Tai HP, Spectra Physics, CA) (53). For the SHG signal detection, an excitation wavelength of 800 nm was used. The backscattered SHG signal has been acquired by a photomultiplier tube (HC125–02, Hamamatsu, Japan), after being collected through a high working-distance objective (NA = 0.95, WD = 2 mm, 20X, water immersion, XLUMPlan FI, Olympus, Japan) and filtered by a 400/20 nm band-pass filter (Chroma Inc., Brattelboro, VT, HQ400/20). The laser polarization has been controlled by a half-wavelength waveplate placed along the optical path.

### Image Acquisition

Sequential images of entire tumor sections have been acquired by rotating the half-wavelength waveplate from 0 to 180° in steps of 5°. An excitation laser power of P_exc_ = 50 mW, measured before the scanning-head, has been exploited for image acquisition of the histology samples. Each image is the result of 3 Kalman average scans and has been acquired in 3.4 s, with a field of view (FOV) dimension of 377 × 377 μm^2^ (512 × 512 pixels). Different FOVs have been stitched by means of the Stitching plugin of the ImageJ software (U.S. National Institute of Health, Bethesda, Maryland, USA) to obtain the mosaic of the entire section.

Both the total tumor sections or ROIs of different sizes (from 75 × 75 to 150 × 150 μm^2^) have been analyzed by means of the μMAPPS software coupled to the cluster algorithm.

### Mouse Models

All mice, inoculated with the tumor cell lines CT26 or 4T1, were BALB/c females of 7–12 weeks of age. The mice were kept in a pathogen-free conventional animal house facility.

The animal house is run by professional employees fully equipped with state-of-the-art instrumentation in order to maintain the standard of animal welfare at the maximum levels. All mice were housed in individual, ventilated cages with 12 h light/dark cycles with food and water *ad libitum*. Experiments were performed using protocols approved by the Institutional Animal Care and Use Committee of the University of Milano-Bicocca and by the Italian Ministry of Health.

### Cells

The BALB/c mouse colon carcinoma CT26 (ATCC, CRL2638) or the triple negative breast cancer 4T1 cell lines were cultured in IMDM-10 complete medium: IMDM, 10% heat-inactivated FBS (EuroClone), 2 mM l-glutamine, 100 U/ml penicillin, 100 μg/ml streptomycin. Cells were collected when the confluence reached the 70%.

### Tumor Injection and Analysis

For the CT26 and 4T1 tumor analysis, BALB/c mice were inoculated in the deep derma in the left flank with the minimal tumorigenic dose of CT26 or 4T1 tumor cells (5 × 10^4^) at Day 0. Explanted tumors at Day 5 were embedded in OCT freezing media (Biooptica). Sections (5 μm) were cut on a Cryostat, adhered to Superfrost Plus slide (Thermo Scientific), and then imaged under the two-photon excitation microscope.

Mice subjected to tumor injection were monitored on a daily basis for signs of discomfort, including hunched posture, ruffled fur and lack of movement within the cage. The body condition score index (a qualitative assessment of an animal overall appearance based on its weight, muscle mass, and bone prominence) was used to evaluate the welfare of the mice. Mice did not present signs of distress given the short period of time between tumor injection and killing.

Three entire tumor sections from three different animals (from 100 to 300 fields of view for each section) have been analyzed for both tumor models. The method has been also applied to five different tumor sections that were acquired as a set of 6 fields of view per sample, encompassing the skin-tumor boundaries.

## Image Processing

### μMAPPS Analysis Method

The μMAPPS method has been reported in Radaelli et al. (39), while a further description of its extension to the analysis of whole histopathology sections can be found in Appendix A.

Here we considered the following microscopic theoretical model for the SHG response *I*($\theta_{L}^{n}$) (31, 54–56):

(1) $$\begin{array}{l} I(\theta_{L}^{n})=k\{\sin^{2}\left[2\left(\theta_{L}^{n}-\theta_{F}\right)\right]+[\sin^{2}(\theta_{L}^{n}-\theta_{F})\\ \;{+\gamma \cos^{2}(\theta_{L}^{n}-\theta_{F})]}^{2}\} \end{array}$$

where $\theta_{L}^{n}$–θ_*F*_ is the relative angle between the laser polarization and the mean collagen fibrils orientations in the image plane (see Appendix A). γ = χ_*zzz*_^(2)^/χ_*zxx*_^(2)^ is the ratio between the entries of the susceptibility tensor ${\underline{\underline{\chi }}}^{\left(2\right)}$, and the scale factor k includes the absolute intensity of the SHG signal as affected by the setup parameters.

The same clustering procedure implemented in Rodriguez et al. (52) has been exploited to obtain clusters of pixels sharing similar microscopic parameters in the entire images or in regions of interest of the tumor sections. This algorithm is based on the maximum density approach and it is applied in the (θ_F_, γ) space. Only clusters characterized by a number of elements higher than a set threshold (ET), computed as a percentage of the total number of analyzed pixels, have been considered. Moreover, the distance between each element belonging to a cluster and its center must be lower than arbitrary chosen cut-off values, named θ_C_ and γ_C_.

We defined the “fibril” entropy in a region of interest as:

(2) $$\begin{array}{r} S=\frac{-\overset{N}{\underset{i=1}{\sum }}p_{i}\log p_{i}}{-\log\frac{1}{E_{C}}}=\frac{-\overset{N}{\underset{i=1}{\sum }}\frac{x_{i}}{E_{C}}\log\frac{x_{i}}{E_{C}}}{-\log\frac{1}{E_{C}}} \end{array}$$

where p_i_ is the probability of occurrence of the i-th cluster measured as the ratio between the number of elements in the i-th cluster and the total number of clustered elements E_C_, and N is the total number of clusters. For a perfectly ordered microstructure, we expect a single cluster, *N* = 1, and p_i_ = 1, therefore S = 0. For a maximally disorder microstructure, instead, we expect N = E_C_, and p_i_ = 1/N. In this case, we retrieve S = 1.

### Software

All the polarization-dependent analysis based on the phasor approach, the θ and γ p-plots, and the θ_F_, γ, clusters, CER, N_C_, and entropy maps have been performed by means of a custom designed C++ based software. The θ and γ histograms have been obtained by means of the software Origin (Origin 8.5, OriginLab Corporation). All the acquired images have been visualized and linearly contrast-adjusted using ImageJ (U.S. National Institute of Health, Bethesda, Maryland, USA) or the Photoshop software.

### Statistical Method

Results are expressed as mean ± SEM. All statistical analyses were performed by GraphPad Prism Software. Means between two groups were compared with a paired two-tailed Student's *t*-test. The degree of significance was assigned as: ^*^p ≤ 0.05 and ^**^p ≤ 0.01, ^***^p ≤ 0.001, and ^****^p ≤ 0.0001. It is noteworthy that, while *p* = 0.01 already accounts for a significant difference between the analysis results, some of the parameters computed on tissues of different morphologies corresponds to even larger significance, up to *p* ≤ 0.0001.

## Results

The efficacy of μMAPPS in characterizing the microscopic properties of the collagen organization and their spatial heterogeneity in extended tumor sections has been tested.

The microscopic θ_F_ and γ parameters, extracted pixel-by-pixel by means of μMAPPS, are here analyzed at a mesoscopic level by characterizing and quantifying their local heterogeneity on a tunable spatial scale (the ROIs dimension) with three p-parameters, whose aim is to maximally discriminate different collagen organization within tissues. In order to select the heterogeneity level we exploit a clustering algorithm (52), defined in the 2D phasor space. The parameters describing the clusters properties (N_C_, CER, and fibril entropy) can be taken as meta-features that could then be combined to conventional morphological features to support the histopathology analysis in a user-independent way.

We report in the following an example of our analysis applied to a simple, but in our opinion meaningful, model in which a tumor tissue is surrounded by a skin layer. The aim of this study is to provide a proof-of-concept of the ability of our method to distinguish among tissue areas characterized by different local properties.

The analysis of the histology section is reported at two levels: at a first level, the tumor and skin areas have been separated and globally analyzed, while the second approach relies on the sequential analysis of regions of interest encompassing the entire section.

**Figure 1:**
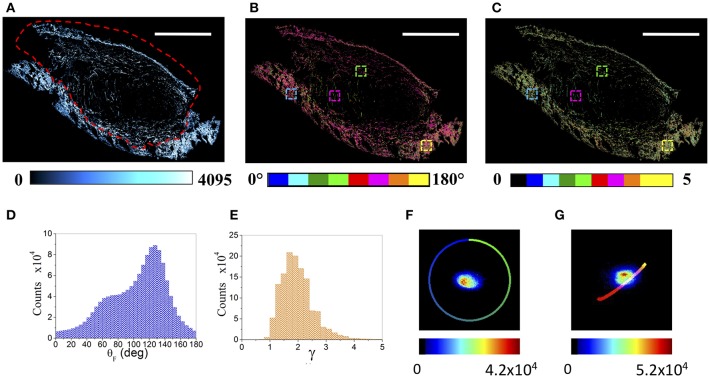
μMAPPS analysis of an entire tumor (CT26) section. **(A)** Maximum intensity projection of the mosaic reconstruction of the entire tumor section. Image size: 3.8 × 2.5 mm^2^. Scale bar: 1 mm. The dashed red line indicates the tumor-skin boundary. **(B,C)** Show the global θ- and γ- maps, color-coded as in the legend. The four colored boxes show the 150 × 150 μm^2^ ROIs reported in the following Figure 4. **(D,E)** Report the corresponding global θ- and γ- counts histograms, while **(F,G)** show the θ- and γ- phasor plots of the entire tumor section, respectively. These results refer to the tumor section shown in **A–C**. The color scale encodes for the counts per phasor plot pixel. The θ reference curve in **(F)** has been obtained by simulating equation (1) within the [0, π] angular range and γ –> ∞, while the γ reference curve in **(G)** has been obtained by simulating equation (1) within the [θ_F_, (θ_F_ +π/2)] angular range while varying γ from 0 to 10.

Figure 1A shows a tumor section (5 μm thickness, 3.8 × 2.5 mm^2^, CT26 derived colon carcinoma from mice, explanted 5 days after cells inoculation), obtained as a tilescan of sequential superimposing images (377 × 377 μm^2^, 512 × 512 pixels each), acquired as a function of the laser polarization.

The collagen composing the skin and the tumor fibers, revealed by the SHG signal, shows two distinct morphologies (Figure 1A). The skin is constituted by high dense basket weaves oriented collagen fibers (57, 58), while the tumor is characterized by wavy, thinner, longer and more sparse fibers. Each pixel has been Fourier transformed (Discrete Fourier Transform, DFT) into a point in two connected phasor plots, as described in Appendix A and in Radaelli et al. (39). The θ_F_ and γ maps related to each single acquired image have been extracted, by exploiting Equations (A3, A4), from the relative θ and γ p-plots, and combined to obtain the resulting color-coded mosaic θ_F_ and γ maps of the entire sample (see Figures 1B,C). Figures 1D,E show the histograms of the θ_F_ and γ values (related to the tumor section in panel A), recovered from the global phasor plots in Figures 1F,G. The anisotropy parameter γ retrieved on the entire tumor section spans a wide range [0.8–4.5] with a monomodal distribution, while the fibrils angle is characterized by a large doubly peaked distribution. An example of the results obtained for the 4T1 tumors is reported in Supplementary Figure 1.

### Separate Analysis of Tumor and Skin Areas

**Figure 2:**
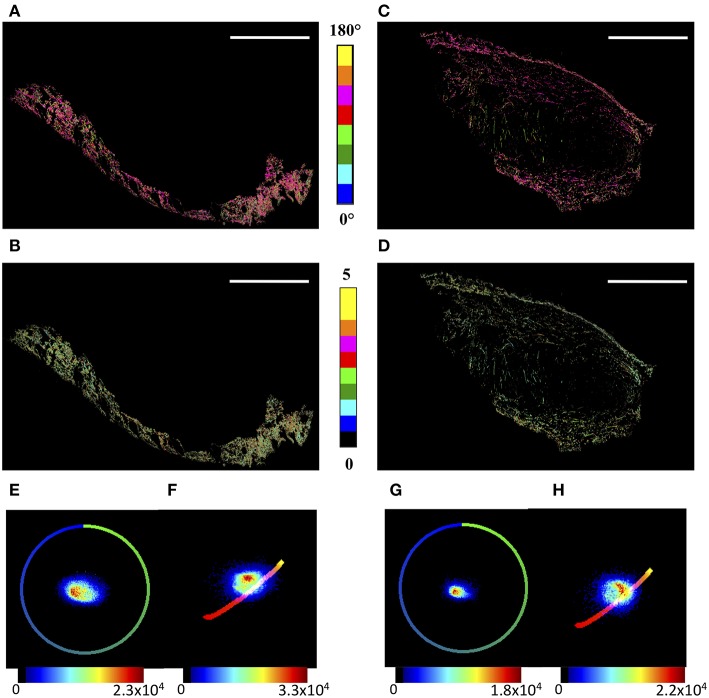
μMAPPS analysis of the skin and tumor regions. **(A,B)** show the θ- and γ- maps, color-coded as in the legend, of the skin region, while **(C,D)** report those of the tumor area. Image size: 3.8 × 2.5 mm^2^. Scale bar: 1 mm. **(E,F)** show the θp-plot and γp-plot of the skin region, while **(G,H)** report those of the tumor area. The plots were exploited to derive the θ- and γ-maps according to Equations (A3,A4). The color scale encodes for the counts per phasor plot pixel.

In order to highlight the global microscopic behavior of tumor and skin regions, their boundaries were manually selected by an expert, based on tissue morphology shown in H&E and PicroSirius Red stained sequential sections. The θ_F_ and γ maps are reported in Figures 2A–D for the skin and the tumor areas, respectively, while the corresponding θ_F_ and γ p-plots are shown in Figures 2E–H.

Moreover, while the angles show wide distributions in both cases, the γ values lie in the range [0.8–3.5] for the tumor, and the skin shows a tail up to γ ≅ 4.5. Correspondingly, the peak value obtained by a gaussian fit of the skin distribution is slightly larger, < γ > = 1.83 ± 0.02, than that of the tumor, < γ > = 1.77 ± 0.01, with a full width at half maximum of the distribution of <FWHM>_skin_ = 0.95 ± 0.05 and <FWHM>_tumor_ = 0.90 ± 0.03, respectively.

#### Clustering Algorithm and p-Parameters

The microstructure in-homogeneity of the tumor and skin regions has been characterized by means of the number and size of the clusters, and how they scale with the choice of the cutoff conditions.

**Figure 3:**
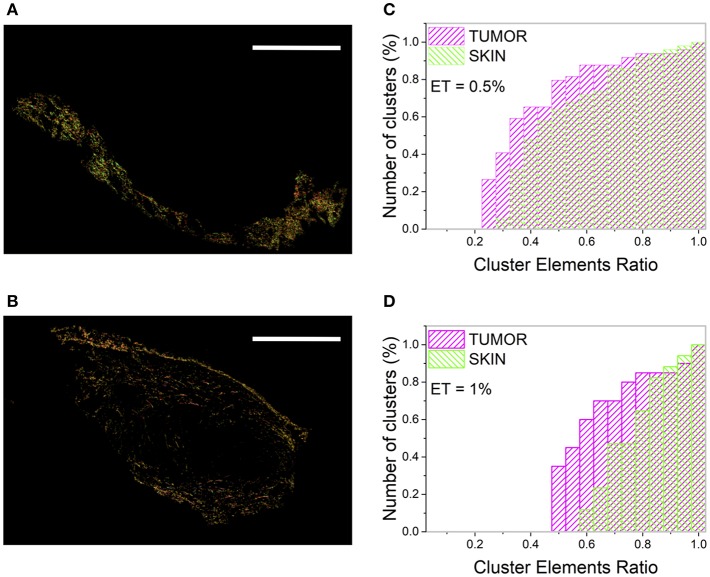
Clustering procedure of the skin and tumor regions. **(A,B)** show the result of the clustering procedure applied separately to the skin **(A)** and the tumor **(B)** area by assuming θ_C_ = 5°, γ_C_ = 0.2, and ET = 1%. Image size: 3.8 × 2.5 mm^2^. Scale bar: 1 mm. Each monochromatic LUT encodes for a cluster. **(C,D)** show the cumulative distribution of the number of clusters (reported as percentage) as a function of the CER for the tumor (magenta) and the skin (green), related to the tumor section in Figure 1A.

To study how the heterogeneity of the tissue microscopic properties (obtained with tight cutoff values: θ_C_ = 5°; γ_C_ = 0.2; ET = 1%) is reflected at large spatial scale, the number of clusters (N_C_) and the Cluster Elements Ratio (CER) distribution have been computed on the two entire regions. At this spatial scale, the skin shows a higher (microscopic) order level with respect to the tumor. In fact, by separately analyzing the skin and the tumor area, 14 and 21 clusters were retrieved, respectively (Figures 3A,B). The ratio N_C_(tumor)/N_C_(skin) spans the range [1.2–1.5] for the CT26 samples, and the range [1.25–1.35] for the 4T1 tumors, and it lowers by reducing the ET value.

The CER cumulative distribution (Figures 3C,D) provides a better visualization of the sample in-homogeneity and of its dependence on the ET value. A higher number of clusters with few elements (small CER) has been retrieved for the tumor with respect to the surrounding skin, characterized instead by clusters with a more uniform population.

The tumor is characterized by a higher number of clusters with small CER with respect to the skin, although this value is strongly dependent on the selected ET. In fact, for the section reported in Figure 1A, ~60% of tumor clusters have a CER value below 0.6, while this percentage is reduced to ~12% for the skin (for θ_C_ = 5°, γ_C_ = 0.2, and ET = 1%, Figure 3C). However, the absolute values of the clusters number and the CER distribution values change substantially for ET = 0.5% (Figure 3D), preventing the development of a robust algorithm based on this parameter and able to discriminate between different tissue regions.

The results related to other analyzed samples are reported in Supplementary Figure 2.

In an effort to quantitatively discriminate between the two tissue regions, we moved to evaluate the “fibril” entropy (S, see Equation 2), which, by its definition, should provide an estimate of the local disorder degree more robust with respect to the choice of the cutoff parameters. When we assume tight clustering cutoff values, θ_C_ = 5°, γ_C_ = 0.2, and ET = 1%, in the two tissue regions, we retrieve in this sample ~16% higher values of S in the tumor collagen (S_tumor_ = 0.25) with respect to the skin (S_skin_ = 0.21). Still, even if this difference is reduced to ~11% for ET = 0.5% (S_tumor_ = 0.36 and S_skin_ = 0.32), among all the analyzed tumor sections, we retrieved a well-defined mean ratio S_tumor_/S_skin_ = 1.21 ± 0.03 for the CT26 samples (ET = 1%). For 4T1 tumor model, the value S_tumor_/S_skin_ = 1.19 ± 0.05 has been obtained.

#### Dependence of the Phasor Parameters on the Clustering Conditions

A systematic investigation of the effect of the choice of the cutoff conditions (θ_C_ = 5° ± 1°, γ_C_ = 0.20 ± 0.02 for ET = 1, 0.7, 0.5%) on the evaluation of the three p-parameters on the two tissue regions is reported in the Supplementary Materials for the tumor in Figure 1A. As shown in Supplementary Figure 3, all the three parameters can discriminate the separate collagen organization in the two macroscopic areas, with more significative results (*p* < 0.001) for N_C_ and S. Similar results have been obtained for all the analyzed samples.

### Sequential Analysis on Regions of Interest With Different Sizes

**Figure 4:**
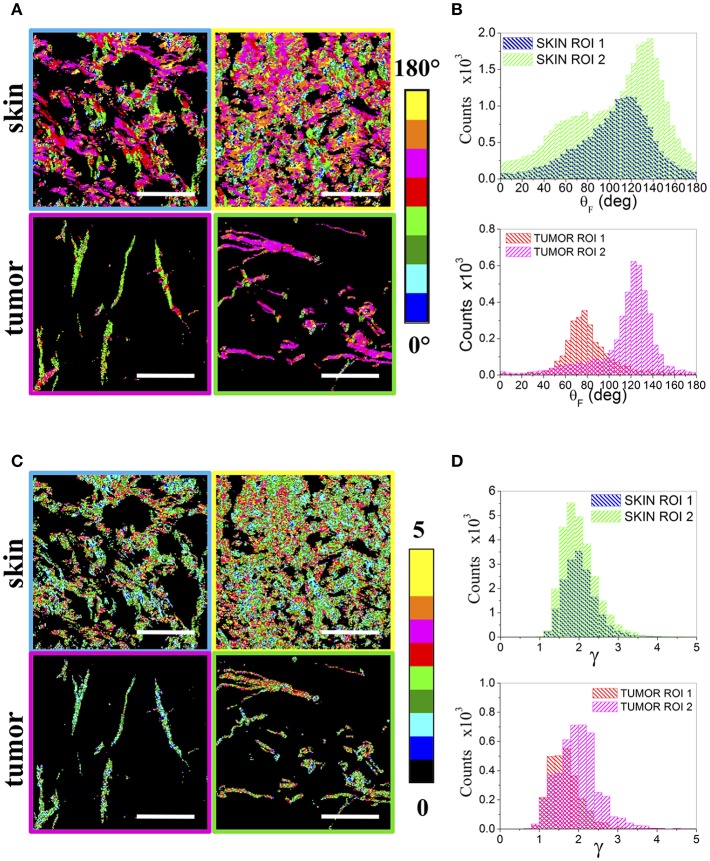
Regions of Interest. **(A)** shows the θ_F_ maps of 150 × 150 μm^2^ ROIs obtained from two skin (above) and two tumor (below) areas, while **(B)** reports the related θ_F_ distribution histograms. The corresponding γ maps are reported in **(C)** for the skin (above) and the tumor (below). The ROIs frames are color coded as the corresponding boxes shown in Figures 1B,C. **(D)** Shows the obtained γ distribution histograms. Scale bar: 50 μm.

Since the boundaries between the two regions are not always sharp and the different collagen organizations can be intertwined, making difficult and user dependent an a-priori area selection, a more refined data analysis has been performed by analyzing sequential non-overlapping ROIs with a size of 75 × 75 and 150 × 150 μm^2^, encompassing the entire tumor section, in order to automatically highlight different local collagen organizations by means of the p-parameters. Figure 4 reports, as an example, the θ_F_ (A) and γ (C) maps for two ROIs, related to the skin and tumor regions indicated in Figure 1A, together with their distribution histograms (B,D). By taking into account all the ROIs, wider peaks for the fibrils angular distributions are mainly obtained in skin regions, while the tumor presents more variable γ distributions with a peak in the range [1.5–2.1].

The clustering procedure has been applied to the θ_F_ and γ values retrieved in each ROI by μMAPPS in order to compute the three p-parameters. Only ROIs where the main cluster contains at least 15 elements for the 75 × 75μm^2^ size, and 30 for the 150 × 150 μm^2^ size, have been considered in the analysis.

#### CER Evaluation

**Figure 5:**
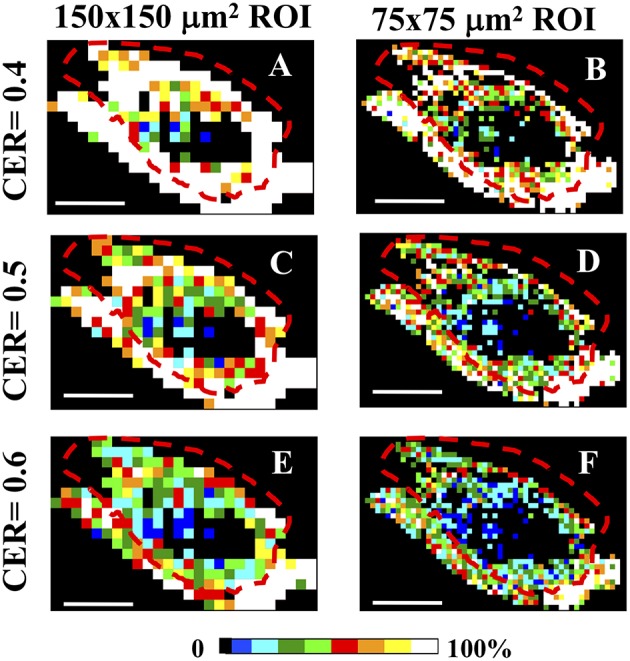
CER analysis in Regions of Interest. **(A–F)** The entire tumor section has been separated in sequential non-overlapping 150 × 150 μm^2^ **(A,C,E)** and 75 × 75 μm^2^ **(B,D,F)** ROIs, encompassing the entire tumor section. Image size: 3.8 × 2.5 mm^2^. Scale bar: 1 mm. Each 150 × 150 μm^2^ and 75 × 75μm^2^ pixels ROI has been color-coded as in the legend to represent the percentage of clusters with a CER = 0.4 **(A,B)**, CER = 0.5 **(C,D)**, and CER = 0.6 **(E,F)** in each ROI. All panels share the clustering cutoff conditions θ_C_ = 5°, γ_C_ = 0.2, ET = 1%. The dashed red lines indicate the tumor-skin boundary.

The color-coded 150 × 150 and 75 × 75 μm^2^ local maps have been computed while varying the cumulative CER and the ET values, by assuming the cutoff conditions θ_C_ = 5°, γ_C_ = 0.2. Although the number of clusters with a specific value of CER is the highest for ROIs belonging mainly to the skin area (coded in white color for ET = 1% and CER = 0.4 for Figures 5A,B, and for CER = 0.5 in Figures 5C,D), this result is highly dependent on the ET and the selected CER values (as shown in Figures 5E,F for CER = 0.6 and ET = 1%), preventing a reliable discrimination of the tumor and skin regions (see also Supplementary Figure 4).

#### Number of Clusters Evaluation

**Figure 6:**
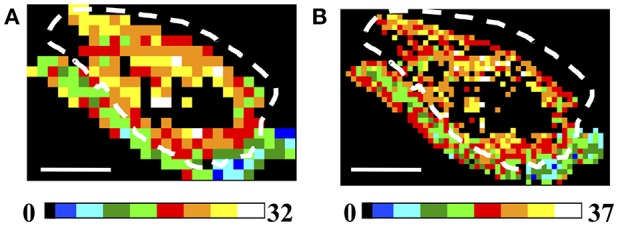
Analysis of Regions of Interest: Number of Clusters (N_C_). **(A,B)** Image size: 3.8 × 2.5 mm^2^. Scale bar: 1 mm. Each 150 × 150 μm^2^ **(A)** and 75 × 75 μm^2^ **(B)** ROI has been color-coded as in the legends to represent the number of clusters retrieved in the ROIs by applying the clustering cutoff conditions θ_C_ = 5°, γ_C_ = 0.2, for ET = 1%. The dashed white lines indicate the tumor-skin boundary.

A more reliable result can be obtained by exploiting the number of clusters. The spatial distribution of the number of clusters is summarized in Figure 6 for low (150 × 150 μm^2^ A) and high (75 × 75 μm^2^ B) spatial sampling, assuming tight clustering conditions (θ_C_ = 5°, γ_C_ = 0.2, ET = 1%). A higher number of clusters is retrieved in the tumor ROIs, also when decreasing the ET (see Supplementary Figure 5). In this case, although the difference between the tumor and the skin areas is smoothed in the N_C_ maps, it is still possible to discriminate between the two regions. Similar results have been obtained in all the analyzed tumor samples (see Supplementary Figure 6 for an example in a 4T1 tumor model sample).

#### Fibril Entropy Evaluation

**Figure 7:**
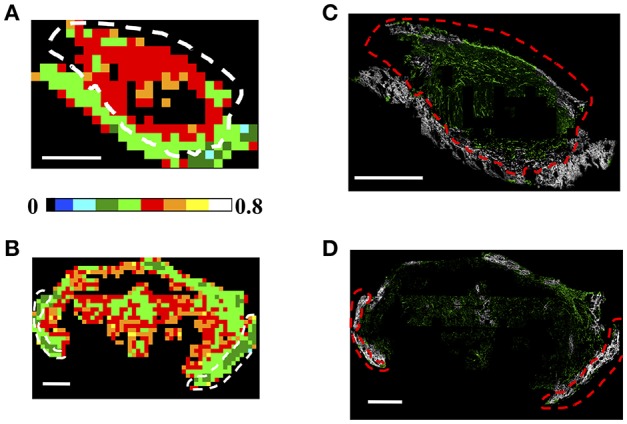
Analysis of Regions of Interest: Fibrils Entropy S. **(A,B)** Each 150 × 150 μm^2^ **(A,B)** ROI has been color-coded as in the legend to represent the retrieved entropy values in a CT26 tumor **(A)** and a 4T1 sample **(B)**, by applying the clustering cutoff conditions θ_C_ = 5°, γ_C_ = 0.2, for ET = 1%. Scale bar: 1 mm. **(C,D)** Results of the segmentation procedure retrieved by backprojecting the entropy information into the image plane for the CT26 **(C)** and the 4T1 **(D)** tumor models. The pixels in the acquired image connected to elements in the ROIs with an-entropy above a selected threshold are shown in green, while those below this threshold are colored in gray. The threshold has been chosen as the mean entropy value computed on the entire section: S_th_ = 0.4 in **(C)** and S_th_ = 0.42 in **(D)**. The dashed white and red lines indicate the tumor-skin boundary.

Finally, the color-coded entropy maps, obtained with tight clustering conditions (θ_C_ = 5°, γ_C_ = 0.2) are reported for both the CT26 and 4T1 cells derived tumors in Figures 7A,B, respectively, (150 × 150 μm^2^ ROI dimension with ET = 1%). In particular, from Figures 7A,B, it can be observed that S is higher (S = [0.4–0.7]) within the tumor region with respect to the skin area and the skin/tumor edges (S = [0.1–0.45]). This result is maintained also at high spatial sampling. The 75 × 75 μm^2^ color-maps in fact show a similar behavior: the tumor entropy is higher with respect to the skin, with values comprised in the range [0.55–0.75] and [0.3–0.5], respectively, for ET = 1% (see Supplementary Figures 7A,B). Other investigated ET conditions are reported in Supplementary Figure 8.

It is noteworthy that, in all the analyzed CT26 and 4T1 derived tumor samples, the skin and tumor areas always correspond to compact patches with a uniform value of the entropy, which is systematically 0.2–0.3 units lower in the skin than in the tumor.

Therefore, we test the possibility to exploit the entropy parameter, fully defined in a phasor space not directly related to the sample morphology, to automatically segment regions characterized by different collagen arrangements, such as in tumor and skin. Figures 7C,D report the back-projection of the entropy information obtained for ET = 1% into the image plane for both tumor models. In the acquired intensity images we discriminated the pixels related to elements in the ROIs characterized by a “fibril entropy” above or below a selected threshold, chosen as the mean value computed in the entire section (with the clustering condition θ_C_ = 5°, γ_C_ = 0.2, ET = 1%). We then assigned them to a monochromatic green or gray LUT in which the intensity codes for the average P-SHG signal in the pixel, and the color was assigned depending on whether the pixel entropy was below or above a selected threshold value S_th_ (S_th_ = 0.4 for Figure 7C; S_th_ = 0.42 for Figure 7D). As shown in the image, the gray color highlights predominantly the skin region, with few ROIs retrieved in the tumor (mainly in the capsule region). Viceversa, the tumor is mainly enlightened by the green LUT, with some exception of the skin area at the section edge, probably due to a reduced number of clusters retrieved in ROIs encompassing the tissue-glass slide boundary. Overall, an accuracy (percentage of correctly retrieved pixels in the tumor or skin areas, obtained by comparing the number of pixels in the entropy-based segmented images with those extracted by an expert operator selection) of (83.0 ± 4.5)% was obtained for the skin area, while the (91.8 ± 4.4)% of the tumor area was correctly retrieved for the CT26 tumor model. For the 4T1 samples we obtained and accuracy of (87.5 ± 3.9)% for the skin region and (91 ± 6)% for the tumor area. Similar results can be achieved by exploiting higher spatial sampling ROIs, as shown in Supplementary Figures 7C,D. For a direct comparison of the obtained segmentation with the H&E and PicroSirius Red stained images (exploited for the tumor-skin separation), the reader can refer to Supplementary Figure 9 (for the CT26 tumor sample reported in Figure 1A) and Supplementary Figure 10 (for the 4T1 tumor sample shown in Supplementary Figure 1).

## Discussion and Conclusions

Recently, image analysis algorithms and processing techniques attracted much attention in the histopathology field, where a rapid and precise disease diagnosis represents an increasing demand (44–49). In parallel, label-free microscopy has recently gained large interest in this field, due to its capability to image cells and tissues by exploiting the intrinsic signal of proteins and molecules, without the need of expensive and time consuming labeling protocols (6–8). However, the application of high resolution optical microscopy methods has been largely limited to demonstrative projects on small fields of view, scarcely relevant for a pathological analysis.

In this framework, we applied our phasor approach, μMAPPS (39), to the analysis of entire fixed tumor sections, commonly exploited in histopathology, to extract the microstructural collagen fibrils angle (θ_F_) and the anisotropy (γ) parameters. This strategy converts huge amount of optical data in dispersion plots on which clustering algorithms can be directly applied to automatically group regions that share similar properties, with the aim to highlight microstructural changes in the tissue leading to pathologies for an early tumor diagnosis and to evaluate the efficacy of a tumor treatment or rescission after surgery (59).

Here, we coupled our 2D phasor algorithm μMAPPS to the computation of novel p-parameters in the phasor space, in order to automatically recover different collagen organizations and their heterogeneity at a mesoscopic spatial scale (the ROI dimension). As a proof of concept, the method has been applied to separate the contribution of tumor collagen from skin by exploiting only microscopic structural properties retrieved in the phasor space. Among the computed parameters, the 'fibrils entropy' has been translated for the first time to the histopathology section analysis field to quantify the microscopic disorder state of clusters characterized by similar fibrils microscopic θ_F_ and γ parameters, and describe the local heterogeneity of the tissues organization.

Fixed sections in which the tumor was surrounded by a skin layer have been selected and analyzed with two parallel approaches. In the first analysis, the tumor has been manually separated from the surrounding skin edges, based on H&E and PicroSirius Red stained sections, to highlight their different microscopic behavior. By applying μMAPPS and the clustering algorithm on the two areas, we retrieved (for ET = 1%) a higher number of clusters in the tumor with respect to the skin, with N_C_(tumor)/N_C_(skin) = 1.34 ± 0.10 for the CT26 tumor models and N_C_(tumor)/N_C_(skin) = 1.30 ± 0.03 in the case of 4T1 derived samples. Moreover, a higher entropy value has been retrieved in the tumor regions, where S_tumor_(CT26) = 0.26 ± 0.03 and S_tumor_(4T1) = 0.30 ± 0.02 with respect to S_skin_(CT26) = 0.22 ± 0.03 and S_skin_(4T1) = 0.26 ± 0.02, obtained in the skin (for θ_C_ = 5°, γ_C_ = 0.2, ET = 1%). The same behavior is observed also for decreasing values of ET, supporting our hypothesis that this parameter can help in separating the two regions in a user independent approach, at least for the two tumor models exploited here.

A more refined and operator-independent analysis has been then performed by automatically separating the entire tumor section in 150 × 150 μm^2^ (and 75 × 75 μm^2^, see Supplementary Materials) non-overlapping windows. In this analysis, μMAPPS and the clustering algorithm have been applied on each ROI to compute the three p-parameters: the percentage of clusters with a selected CER, the number of clusters and the “fibril entropy.” We have tested the dependence of the results on the clustering procedure, the selected thresholds and the ROI dimension, and assessed to what extent we can use the p-parameters to automatically highlight the different local behavior of tumor and skin collagen and which is the most effective one.

The percentage of clusters with a CER ≤ 0.6 is an effective parameter in distinguishing the tumor from the skin when an ET = 1% is assumed. However, this difference decreases when the ET is reduced. Furthermore, tumor ROIs are characterized by a higher number of clusters with respect to the skin, with N_C_ values comprised in the range [15–25] for the CT26 tumors and [20–25] for the 4T1 samples (when θ_C_=5°, γ_C_=0.2, ET=1%), compared to the range of N_C_ of [15-20] in the skin. We also noticed that the entropy is 0.2–0.3 units higher in the tumor ROIs than in the skin. Also N_C_ and the entropy are somehow affected by the choice of the threshold ET. However, the difference of these two parameters on the two morphological area remains significative, irrespective of the ET value, and can be used to discriminate the two regions and to highlight the tumor edges. In this regard we showed (Figure 7) that the segmentation of the two regions obtained by exploiting the entropy information (ET=1%) agrees with the manual segmentation with an accuracy (computed by considering both tumor and skin areas) of 87.4 ± 3.8% for the CT26 and 89.8 ± 5.3% for the 4T1 tumor models.

We believe that these results are particularly encouraging in the direction of an automated algorithm that, by exploiting non-morphological features, will assist pathologists for a fast and reliable diagnosis. However, a number of additional tests and benchmarks are in order and some limitations need to be discussed.

First of all, the computation of the θ_F_ and γ parameters was based on a specific, though widely adopted (31, 54, 55) molecular model. Under the assumption of cylindrical and Kleinmans symmetry conditions, from the point of view of the retrieving algorithm, both the 2D and the 3D models (i.e., in plane or out of plane molecular arrangements) reduce to Equation (1) (60).

Depending on the theoretical model a-priori assumed for the specific tissue investigated, the parameter γ assumes a different interpretation (31) since it can be related to specific molecules and fibrils arrangements (fibrils with uniform alignment, bundles of aligned or tilted fibrils). Since our aim is to discriminate between two different collagen arrangements in two different tissues in an operator-independent way, in this manuscript we did not derive microscopic geometrical parameters of the fibrils and collagen molecules inside a pixel (61–63). Instead, we introduce a statistical parameter, the “fibrils entropy,” to explore the relation between both the θ and γ values on a mesoscopic spatial scale (the ROI dimension) to automatically quantify the local heterogeneity of the tissues organization, based on the simplest model available in the literature.

However, by exploiting the a-priori knowledge of the theoretical model describing a particular tissue (31), we could further evaluate NC, CER, and S on the fibrils and molecules microscopic arrangements, retrieved from the γ values. Moreover, refined models which take into account multiple components of the susceptibility tensor [see for example models in references (27, 28, 60, 61, 64–67)] could reveal different behavior in terms of NC, CER, and S.

For a fully exploitation in the pathology field, we need to validate our method on additional tumor models and alternative tissue sections preparations. To this aim, formalin fixed paraffin embedded sections of human tumors, already tested in P-SHG imaging (68), will be extensively investigated in our lab in order to understand if the entropy based discrimination algorithm could be advantageously applied to differentiate between tumorous and healthy regions within the same tissue type.

Regarding extension of our SHG phasor algorithm, we notice that other methods devoted to pure intensity-based image analysis, showing different collagen fibers organization among healthy and pathological tissues have been proposed. These methods quantify collagen organization from the SHG images by means of 2D Fast Fourier Transform (69, 70), wavelet transform (71), quantification of fiber structure and alignment (72–75), fractal analysis (76), texture analysis of SHG images using first-order and second-order statistics (e.g., gray-level co-occurrence matrix (GLCM) (77–79), or a combination of them (80). We think that the method proposed in this manuscript could be coupled both to intensity-based image analysis algorithms and to other p-parameters, defined also in other phasor spaces, such as those based on the autofluorescence spectral and lifetime decomposition (32, 33), in order to expand the dictionary of features available for pathologic tissue characterization and therefore help physicians during diagnosis, especially for diseases characterized by a large heterogeneity not only between patients, but even within the same tissue specimen.

Furthermore, for an application of our 2D phasor method to thick biopsies, a fact that will dramatically simplify the tissue preparation procedure, the “fibril entropy” based threshold segmentation should be extended to a 3D analysis. This is particularly relevant because collagen fibers create different 3D ECM organization in tumors, depending on the tumor model and on the stage of development. Therefore, the extensive characterization of the collagen 3D structure, also from the microscopic point of view, is of fundamental importance to extract features that can be exploited for diagnosis.

As for the present application to the CT26 and 4T1 tumors inoculated in mice, we have demonstrated that our approach offers multiple advantages: it is able to perform fast microstructural analyses in entire tumor sections that could assist pathologists for a timely and precise disease diagnosis. It is cost-effective since it is based on label free microscopy: exogenous fluorescent dyes are not necessary to elicit second harmonic generation signal, which instead is a non-linear coherent optical process where two incident photons of frequency ω are converted into a single photon of exactly twice the frequency and it is related to the intrinsic symmetric properties of molecules (e.g., collagen).

Our approach is based on non-linear optics scanning microscopy, which has superb capabilities to exploit endogenous optical properties of tissues (emitted intensity but also excited state lifetime or non-linear scattering polarization) and to provide optical sections (virtual non-invasive biopsies *in-vivo*) of the tissular architecture that can be coupled to traditional histopathology approaches with deep implications for resolving pathological cues.

In summary, the proposed 2D phasor approach to the label-free second harmonic generation microscopy of collagen ECM, coupled to the definition of the “fibril entropy” parameter, is promising for the long-term goal of this project. We believe that automated analysis algorithms, coupled to label-free non-linear microscopy and Fourier image processing, could represent a viable solution to assist the pathologists' interpretation of data, speeding up the analysis at reduced costs, meeting directly one of the urgent needs of our society.

## Ethics Statement

Experiments were performed using protocols approved by the Institutional Animal Care and Use Committee of the University of Milano-Bicocca and by the Italian Ministry of Health.

## Author Contributions

LS and GC conceived the method and the experiments, and supervised the project. LS, GC, LD, and MC wrote the manuscript. RS modified the previous μMAPPS version and wrote the C++ codes. RS, LS, MB, MC, and LD acquired and analyzed data. FM performed tumor-related preparations and experiments. LS, GC, and FG provided financial support for the experiments.

### Conflict of Interest Statement

The authors declare that the research was conducted in the absence of any commercial or financial relationships that could be construed as a potential conflict of interest.

## Figures and Tables

**Figure A1:**
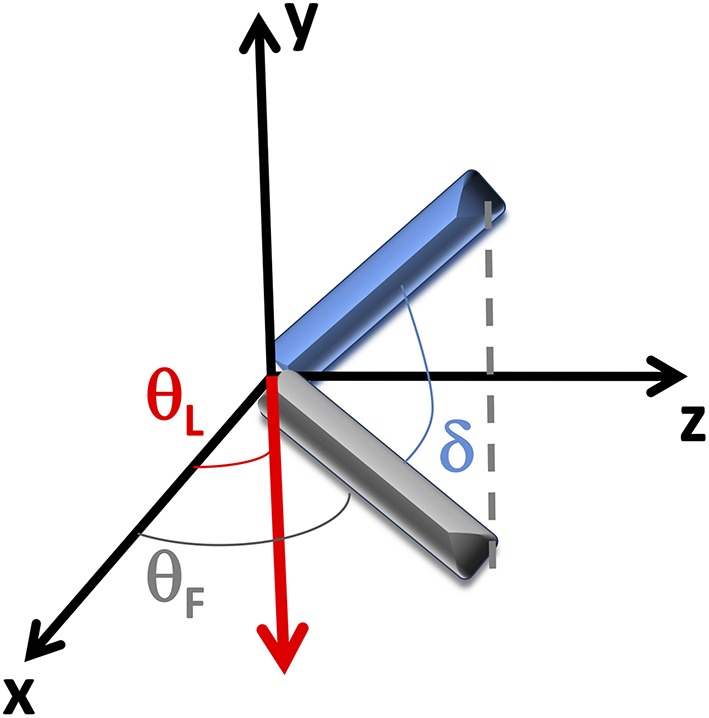
Reference system. Coordinate system scheme: the laser is incident along the y-direction, while the tissue section is positioned in the xz plane. The bundle of fibrils (in blue) lies out of the xz plane with an angle δ. θ_F_ represents the mean angle between the fibrils projection (in gray) into the xz plane and the x-axis, while θ_L_ is the angle between the laser polarization (in red) and the x-axis.

## Appendix A

### Image Processing

#### μMAPPS Analysis Method

The P-SHG signal of each pixel of a stack of images acquired as a function of the laser polarization θ_L_ (Figure A1) is projected onto a first complex plane (θp-plot) via the first harmonic normalized Discrete Fourier Transform (DFT) applied to the 0 ≤ θ_L_ < π range. In the θp-plot, each pixel is transposed into a point, whose (g_θ_, s_θ_) coordinates are:

(A1) $$\begin{array}{l} g_{\theta }=\frac{\sum_{n=0}^{N-1}I\left(\theta_{L}^{n}\right)\cos\left(\theta_{L}^{n}K_{\theta }\right)}{\sum_{n=0}^{N-1}I\left(\theta_{L}^{n}\right)};s_{\theta }=\frac{\sum_{n=0}^{N-1}I\left(\theta_{L}^{n}\right)\sin\left(\theta_{L}^{n}K_{\theta }\right)}{\sum_{n=0}^{N-1}I\left(\theta_{L}^{n}\right)} \end{array}$$

where {*I*($\theta_{L}^{n}$)}_n = 0…N−1_ is the SHG intensity acquired as a function of θ_L_ (0 ≤ θ_L_ <3π/2) with resolution Δθ; K_θ_ = 2π (N) Δθ)^−1^and N = π/Δθ. The angular position of the points in the θp-plot depends only on the collagen fibrils angle θ_F_.

To extract the microscopic order parameter γ, a second interconnected phasor plot (γp-plot) is computed by DFT of the P-SHG in the domain θ_F_ ≤ θ_L_ < θ_F_ + π/2. The coordinates (g_γ_, s_γ_) of the points in the γp-plot are:

(A2) $$\begin{array}{lll} g_{\gamma } & = & \frac{\sum_{n=0}^{N/2-1}I\left(\theta_{L}^{n}+\theta_{F}\right)\cos\left(\left(\theta_{L}^{n}+\theta_{F}\right)K_{\gamma }\right)}{\sum_{n=0}^{N-1}I\left(\theta_{L}^{n}+\theta_{F}\right)};\\ s_{\gamma } & = & \frac{\sum_{n=0}^{N/2-1}I\left(\theta_{L}^{n}+\theta_{F}\right)\sin\left(\left(\theta_{L}^{n}+\theta_{F}\right)K_{\gamma }\right)}{\sum_{n=0}^{N-1}I\left(\theta_{L}^{n}+\theta_{F}\right)} \end{array}$$

where K_γ_ = 2π[*N*(Δθ + θ_*F*_)]^−1^.

To extract the θ_F_ and γ values we took advantage of two reference curves obtained for the θp-plot and γp-plot by considering the microscopic theoretical model for the SHG response described by Equation (1).

The fibril angle θ_F_ is half the value of the angle that the vector pointing from the center of the θp-plot to the point (g_θ_, s_θ_) makes with the phasor plot real axis and it is obtained according to the following expression:

(A3) $$\begin{array}{l} \cos\left(2\theta_{F}\right)=\frac{g_{\theta }}{\sqrt{g_{\theta }^{2}+s_{\theta }^{2}}} \end{array}$$

The parameter γ can been retrieved from the minimum Euclidean distance projection onto the Reference Curve (RC):

(A4) $$\begin{array}{lll} d_{e-RC} & = & \sqrt{{\left(g_{e}-g_{RC}\right)}^{2}+{\left(s_{e}-s_{RC}\right)}^{2}}\\ \gamma_{e} & = & {\gamma_{RC}|}_{\min\left(d_{e-RC}\right)} \end{array}$$

where the minimum is obtained over the value of the γ p-plot reference curve points, computed by exploiting the experimental sampling angle Δθ and θ_F_ measured on the first phasor plot.

The original μMAPPS software has been modified here in order to sequentially analyze the set of images related to the entire tumor sections. The θ p-plot and γ p-plot are computed by DFT of the P-SHG signal of each pixel in the images constituting the mosaic. The color-coded θ_F_ and γ maps are then retrieved from the phasor plots for each image by exploiting Equations (A3, A4). Finally, the θ_F_ and γ maps of the entire tumor section are obtained by mosaic combining the data related to each single image. For features of the tumor represented in two adjacent images, the mean of the θ_F_ and γ values has been computed. The software computes also the global θ p-plot and γ p-plot of the entire tumor section, from which it is also possible to infer differences in the geometrical features related to the microscopic tissue behavior. Only P-SHG spectra above a threshold (T_NL_, reported in the figure captions) are analyzed in order to discard the background contribution. To further reduce the noise, a gaussian filter of the image stack acquired as function of θ_L_ has been implemented before μMAPPS analysis. Moreover, a custom implemented median filter with a 3 × 3 pixels mask was directly and independently applied once to the *s* and *g* coordinates, to reduce the dispersion of the points in the θp-plot and γp-plot.

By means of a nearest neighbor clustering algorithm (52), applied to the two coupled phasor spaces, we could automatically group pixels characterized by θ_F_ and γ values comprised within selected cutoffs (θ_C_ and γ_C_). The total number of clustered pixels and of clusters was limited by the element threshold, ET, that is the minimum number of elements in a cluster with respect to the total number of analyzed pixels.

The software provides also the clusters, CER, N_C_ and entropy maps of the entire tumor sections or related to the different ROI sizes.
