# Supplementary material for: Whole-Section Tumor Micro-Architecture Analysis by a Two-Dimensional Phasor-Based Approach Applied to Polarization-Dependent Second Harmonic Imaging
Source: Front Oncol. 2019 Jun 19;9:527. doi: 10.3389/fonc.2019.00527 (PMC6593899; doi:10.3389/fonc.2019.00527)
Supplement: Supplementary file 1 [file Data_Sheet_1.pdf]

Supplementary Materials

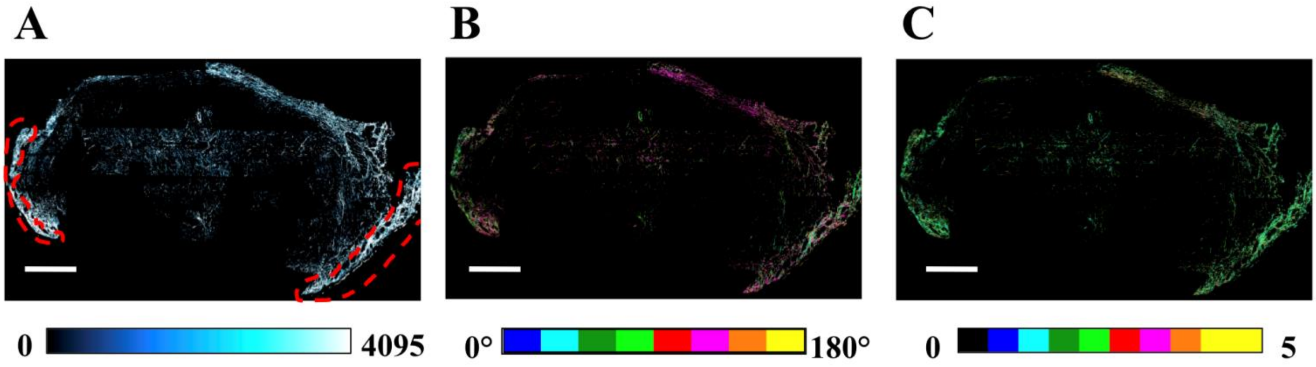

Supplementary Figure 1. **µMAPPS analysis of an entire 4T1 tumor section.** (**A**) Maximum intensity projection of the mosaic reconstruction of the entire tumor section. Image size: 8x5 mm^2. Scale bar: 1 mm. The dashed red line indicates the tumor-skin boundary. (**B**) and (**C**) show the global θ- and γ- maps, color-coded as in the legend.

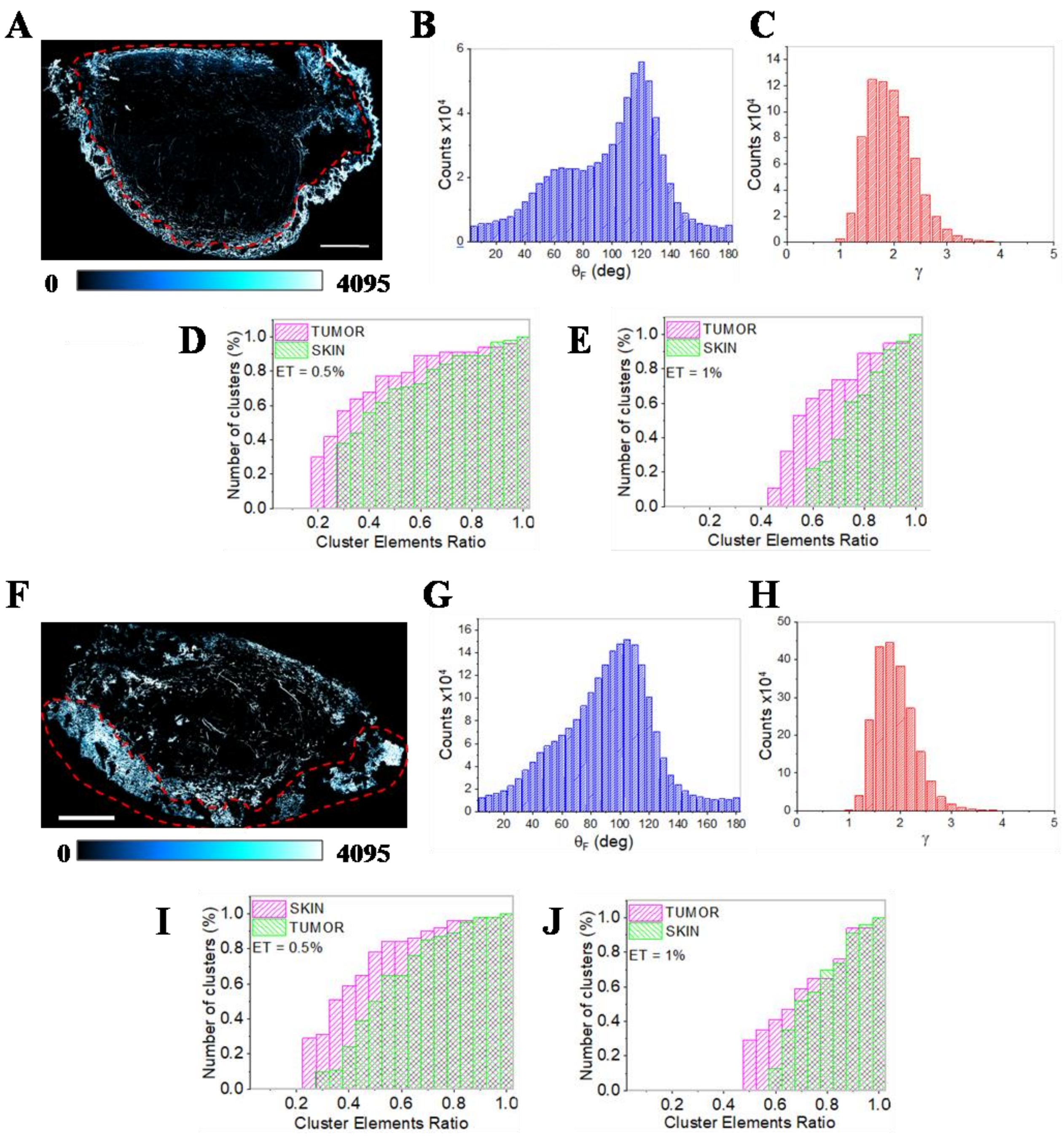

Supplementary Figure 2. **Analysis of further CT26 tumor samples.** (**A**) and (**F**) Maximum intensity projection of the mosaic reconstruction of two entire CT26 tumor sections, related to two different mice. Images size: 4.7x3 mm^2 (panel A); 4.5x2.5 mm^2 (panel F). Scale bar: 1 mm. The dashed red line indicates the tumor-skin boundary. (**B**) and (**C**) show the θ- and γ- counts histograms related to panel A, while (**D**) and (**E**) report the cumulative distribution of the number of clusters (as percentage) as a function of the CER for the tumor (magenta) and the skin (green), for the CT26 tumor section in panel A. (**G**) and (**H**) show the θ- and γ- counts histograms related to panel F, while (**I**) and (**J**) report the cumulative distribution of the number of clusters (as percentage) as a function of the CER for the tumor (magenta) and the skin (green), for the CT26 tumor section in panel F.

Supplementary Note 1

Dependence of the phasor parameters on the clustering conditions.

We performed a systematic search for the effect of the choice of the cutoff conditions on the evaluation of the three p-parameters, defined in the phasor space: the CER, the number of clusters and the overall entropy values on the two tissutal regions. The clustering procedure was applied to the θ_F and γ parameters extracted from the tumor section in Figure 1A by varying slightly the cutoff conditions: θ_C = 5°±1°, γ_C = 0.20±0.02 for ET=1%, 0.7%, 0.5% and the results are summarized in **Supplementary Figures 3A-K** for the most relevant findings. The data are grouped according to the ET value and analyzed by means of the GraphPad Prism software. In particular, the data have been compared by means of a two-tailed paired t-test and the statistical significance level is reported in **Supplementary Figures 3A-K** for the CER (panels A-B; only non-null values have been analyzed), the number of clusters (panels C-D) and the entropy (panels E-F). As shown in **Supplementary Figure 3**, all the three parameters can discriminate the separate microscopic behavior of tumor and skin, with the number of clusters and entropy showing higher significant results for all the exploited ET values.

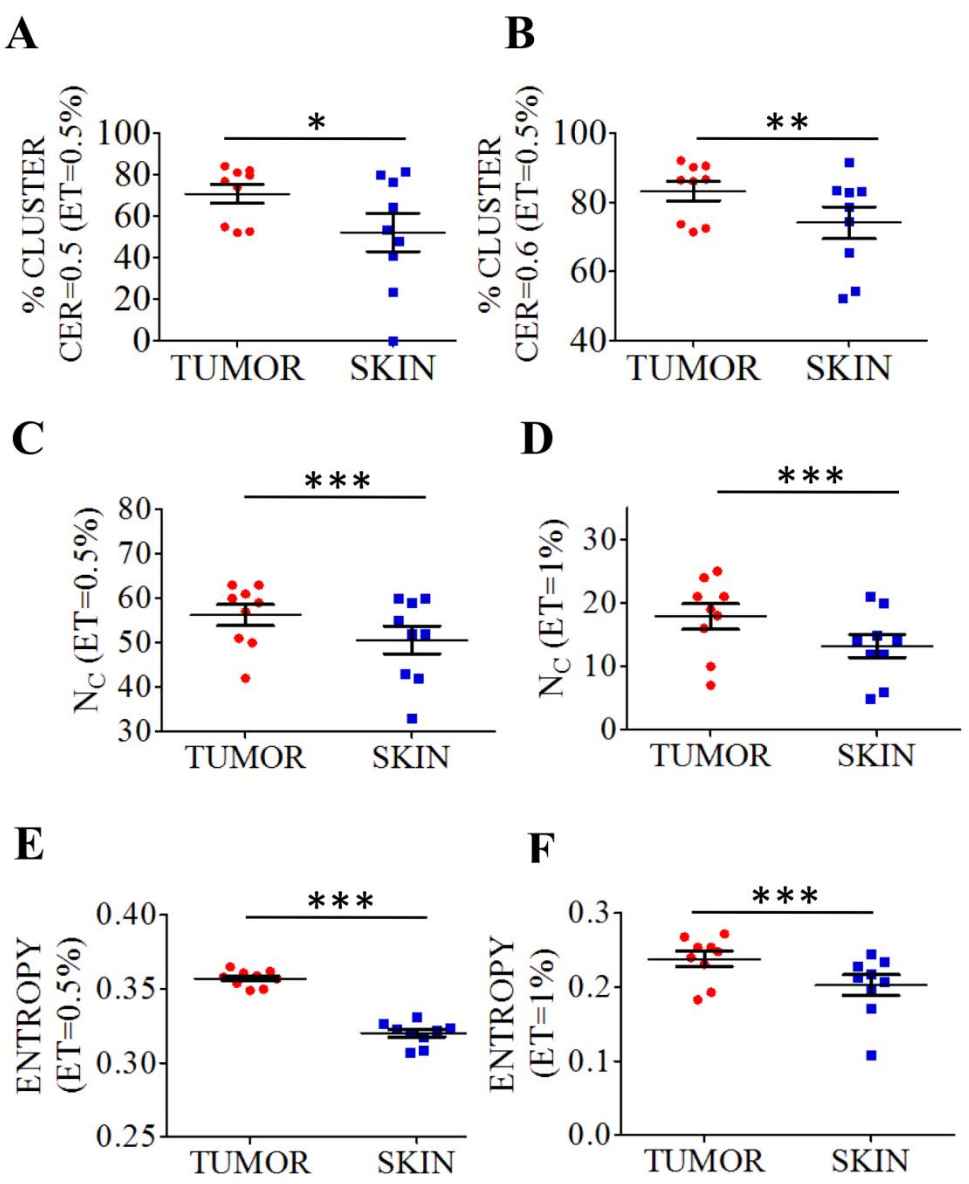

Supplementary Figure 3. **Results of the clustering procedure: microscopic parameters describing the skin and tumor regions.** The data are obtained by exploiting different clustering cutoff conditions: $\theta_C = 5°\pm1°$, $\gamma_C = 0.20\pm0.2$. Data related to each separate ET have been grouped and analyzed through the GraphPad Prism software, while null values have been discarded. (**A**)-(**B**) show the graphs of the results related to the percentage of clusters, retrieved from the tumor in Figure 1A, characterized by ET=0.5% and CER=0.5 (panel A) or CER=0.6 (panel B). (**C**)-(**D**) report the number of clusters in the tumor and skin areas, retrieved for ET=0.5% (panel C) and ET=1% (panel D). (**E**)-(**F**) refer to the fibrils entropy values, computed by Equation (2) for the tumor and skin regions, by applying ET=0.5% (panel E) and ET=1% (panel F). Note that a single asterisk (*) has the meaning $0.01 < p < 0.05$, based on paired two-tailed Student's t-test. Double asterisks (**) denote $0.001 < p < 0.01$, while for a triple asterisks (***) $p<0.001$.

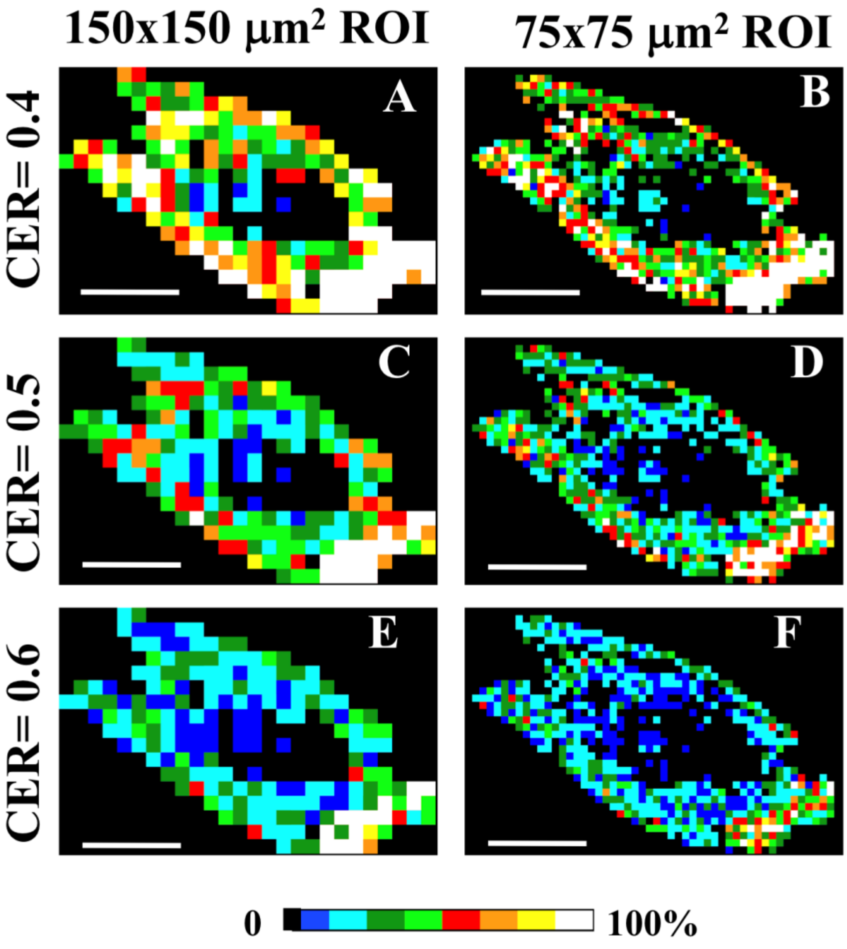

Supplementary Figure 4. **CER analysis in Regions of Interest.** (**A**)-(**F**) The entire tumor section has been separated in sequential non overlapping 150x150 μm^2 (panels A, C, E) and 75x75 μm^2 (panels B, D, F) ROIs, encompassing the entire tumor section. Image size: 3.8x2.5 mm^2. Scale bar: 1 mm. Each 150x150 μm^2 and 75x75 μm^2 pixels ROI has been color-coded as in the legend to represent the percentage of clusters with a CER=0.4 (panels A, B), CER=0.5 (panels C-D) and CER=0.6 (panels E-F) in each ROI. All panels share the clustering cutoff conditions $\theta_C = 5°$, $\gamma_C = 0.2$, ET=0.7%. The discrimination of the tumor area from the skin region on the basis of a threshold on the percentage of pixels with a specific CER value would be substantially affected by the chosen value of CER and ET. In particular, it is more difficult to separate the skin and the tumor regions the lower is the ET value and the higher is the CER over which we project the distributions.

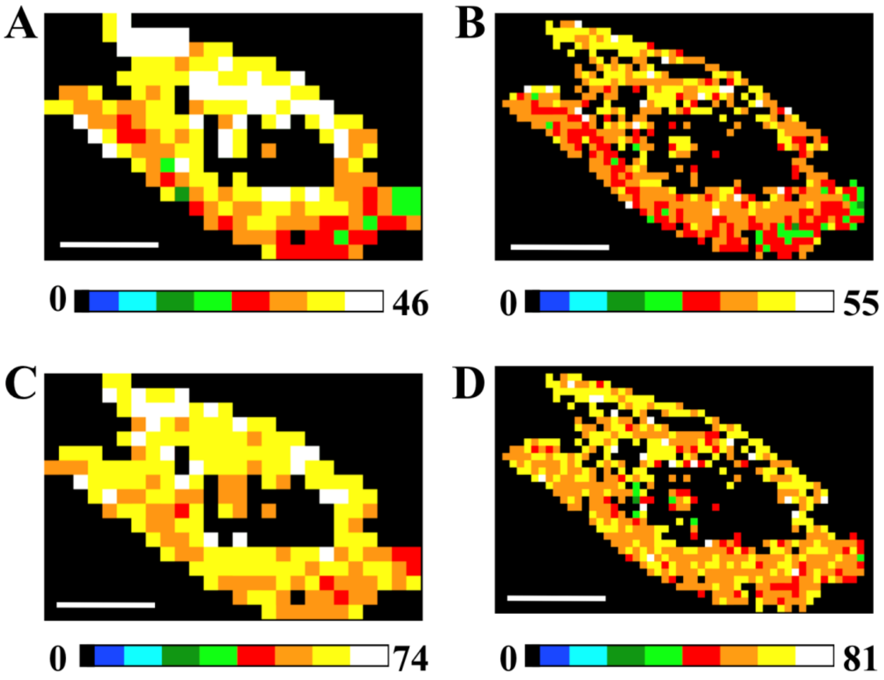

Supplementary Figure 5. **Analysis of Regions of Interest: Number of Clusters (N_C) in CT26 samples**. (**A**)-(**D**) Image size: 3.8x2.5 mm^2. Scale bar: 1 mm. Each 150x150 μm^2 (panels A, C) and 75x75 μm^2 (panels B, D) ROI has been color-coded as in the legends to represent the number of clusters retrieved in the ROIs by applying the clustering cutoff conditions $\theta_C = 5°$, $\gamma_C = 0.2$, for ET=0.7% in panels A-B and ET=0.5% in panels C-D. While decreasing the ET, the N_C increases, since clusters with a lower number of elements are included in the clustering process.

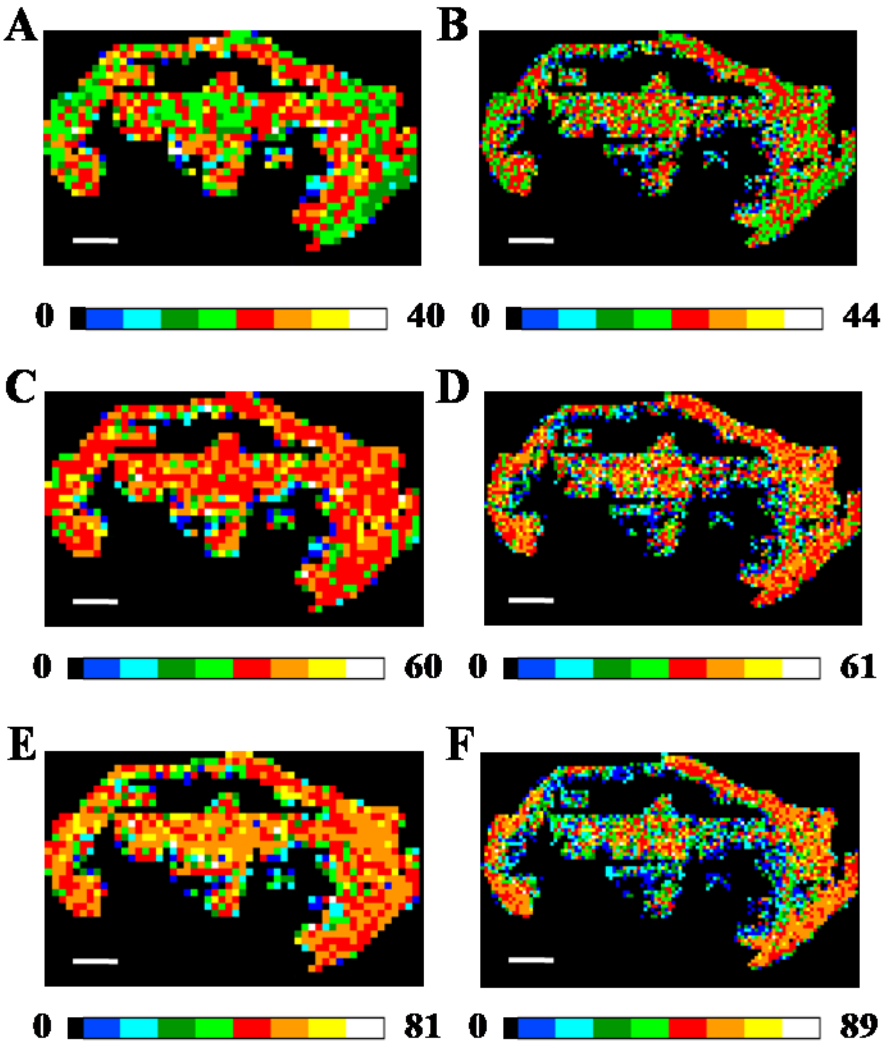

Supplementary Figure 6. **Analysis of Regions of Interest: Number of Clusters (N_C) in 4T1 samples**. (**A**)-(**F**) Image size: 8x5 mm^2. Scale bar: 1 mm. Each 150x150 μm^2 (panels A, C, E) and 75x75 μm^2 (panels B, D, F) ROI has been color-coded as in the legends to represent the number of clusters retrieved in the ROIs by applying the clustering cutoff conditions $\theta_C = 5°$, $\gamma_C = 0.2$, for ET=1% in panels A-B, ET=0.7% in panels C-D and ET=0.5% in panels E-F.

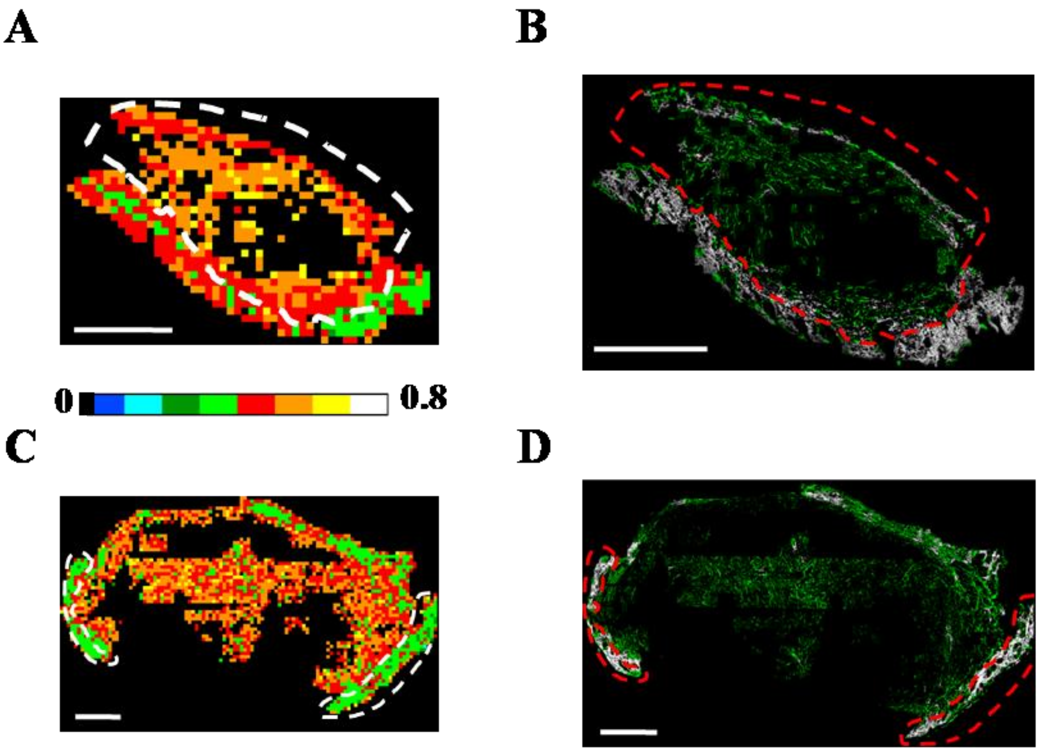

Supplementary Figure 7. **Analysis of Regions of Interest: Fibrils Entropy S**. (**A**)-(**B**) Each 75x75 μm^2 (panels A-B) ROI has been color-coded as in the legend to represent the retrieved entropy values in a CT26 tumor (panel A) and a 4T1 sample (panel B), by applying the clustering cutoff conditions $\theta_C = 5°$, $\gamma_C = 0.2$, for ET=1%. Scale bar: 1 mm. (**C-D**) Results of the segmentation procedure retrieved by backprojecting the entropy information into the image plane for the CT26 (panel C) and the 4T1 (panel D) tumor models. The pixels in the acquired image connected to elements in the ROIs with an entropy above a selected threshold are shown in green, while those below this threshold are colored in grey. The threshold has been chosen as the mean entropy value computed on the entire section: S_{th}=0.48 in panel C and S_{th}=0.45 in panel D. The dashed white and red lines indicate the tumor-skin boundary.

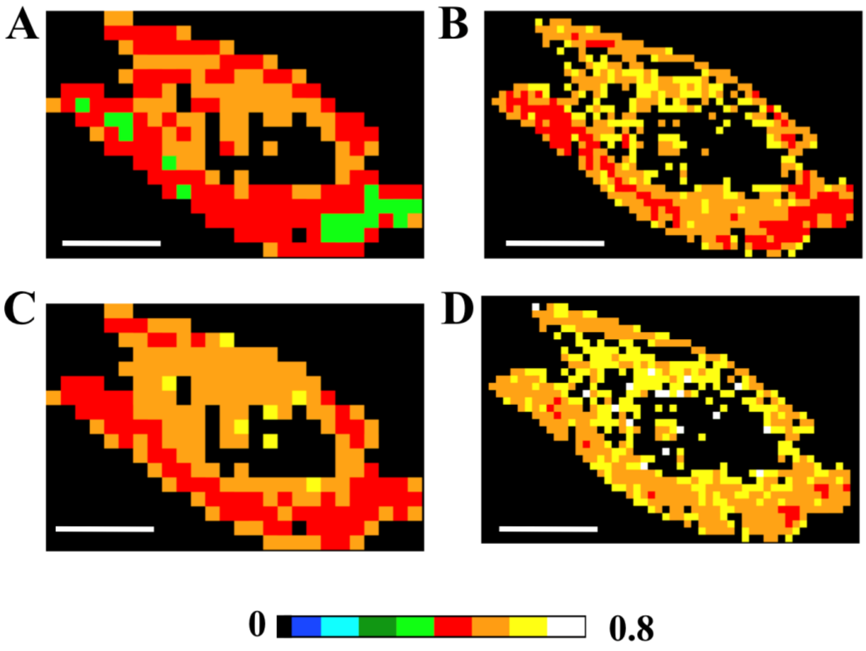

Supplementary Figure 8. **Analysis of Regions of Interest: Fibrils Entropy S**. (**A**)-(**D**) Image size: 3.8x2.5 mm^2. Scale bar: 1 mm. Each 150x150 μm^2 (panels A, C) and 75x75 μm^2 (panels B, D) ROI has been color-coded as in the legend to represent the entropy values retrieved in the ROIs by applying the clustering cutoff conditions $\theta_C = 5°$, $\gamma_C = 0.2$, for ET=0.7% (panels A-B) and ET=0.5% (panels C-D).

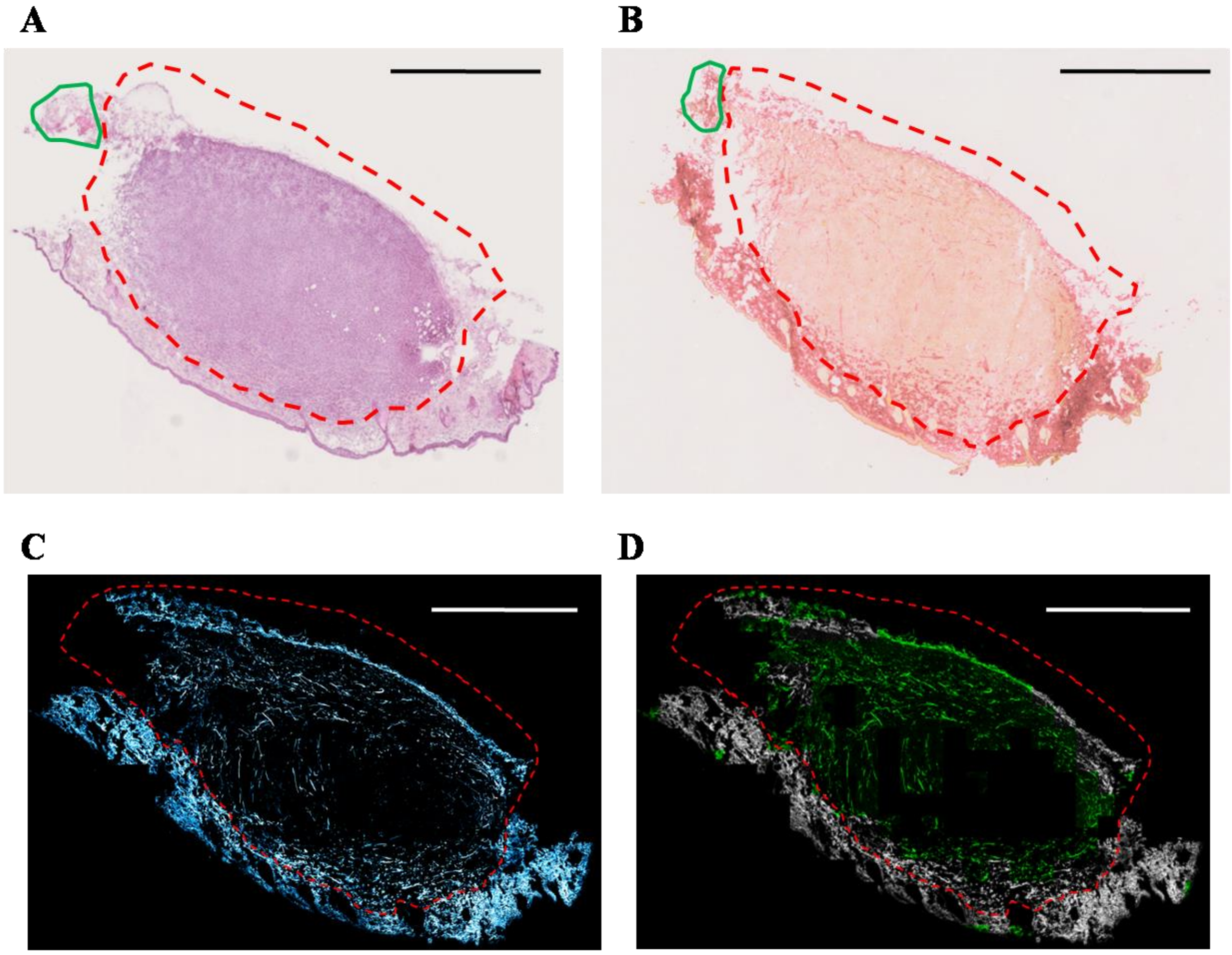

Supplementary Figure 9. **Example of a CT26 tumor section analysis. (A)-(B)** H&E (panel A) and PicroSirius Red (panel B) staining of sequential tumor sections, exploited by three experts to separate the skin and the tumor areas, as indicated by the dashed red line. The tumor region is inside the dashed boundary, while the skin is outside. The green profile indicates an area discarded in the P-SHG analysis due to the uncertainty to its assignment as tumor or skin area. The same separation has been exploited in the SHG image reported in panel (**C**), which shows the maximum intensity projection of the mosaic reconstruction of the entire tumor section, obtained by superimposing sequential 377x377μm^2 (512x512 pixels) images, acquired as a function of the laser polarization ($\Delta\theta$ = 10°). Image size: 3.8x2.5 mm^2. Scale bar: 1 mm. The image is the same reported in Figure 1A in the main text. Panel (**D**) reports the results of the segmentation procedure retrieved by back-projecting the entropy information obtained for ET=1% into the image plane in the case of 150x150 μm^2 spatial sampling area. The pixels in the acquired image connected to elements in the ROIs with an entropy above a selected threshold (S_{th}=0.4) are shown in green, while those below this threshold are colored in grey. The image is the same reported in Figure 7C in the main text.

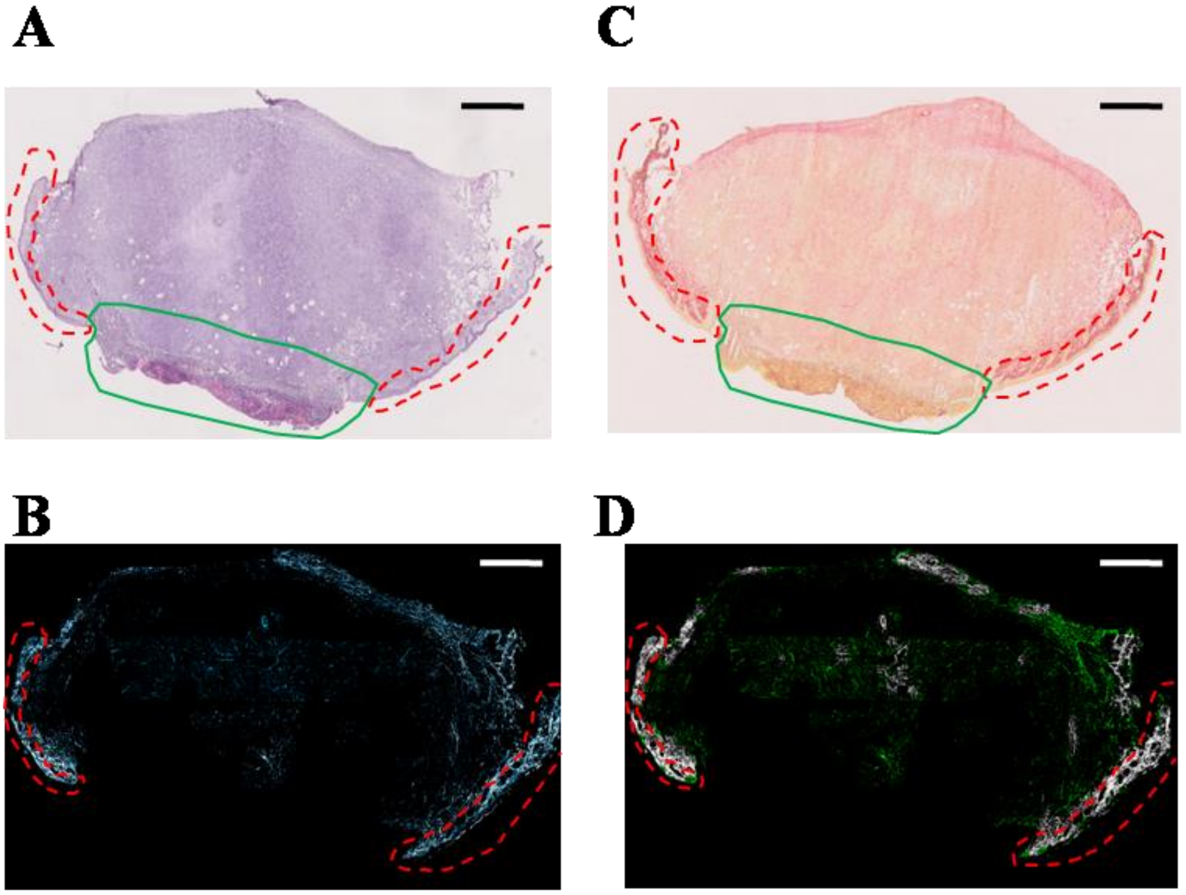

Supplementary Figure 10. **Example of a 4T1 tumor section analysis. (A)-(B)** H&E (panel A) and PicroSirius Red (panel B) staining of sequential tumor sections, exploited by three experts to separate the skin and the tumor areas, as indicated by the dashed red line. The tumor region is inside the dashed boundary, while the skin is outside. The green profile indicates a necrotic area, discarded in the P-SHG analysis. The same separation has been exploited in the SHG image reported in panel (**C**), which shows the maximum intensity projection of the mosaic reconstruction of the entire tumor section, obtained by superimposing sequential 377x377μm^2 (512x512 pixels) images, acquired as a function of the laser polarization ($\Delta\theta$ = 10°). Image size: 8x5 mm^2. Scale bar: 1 mm. The image is the same reported in Supplementary Figure 1A. Panel (**D**) reports the results of the segmentation procedure retrieved by back-projecting the entropy information obtained for ET=1% into the image plane in the case of 150x150 μm^2 spatial sampling area. The pixels in the acquired image connected to elements in the ROIs with an entropy above a selected threshold (S_{th}=0.42) are shown in green, while those below this threshold are colored in grey. The image is the same reported in Figure 7D in the main text.
